# On the path to setting meaningful goals in the care for mental health patients: initial results of the adaptation of Mental Health Quality of Life Questionnaire (MHQoL) in a Hungarian clinical and non-clinical sample

**DOI:** 10.1192/j.eurpsy.2025.2002

**Published:** 2025-08-26

**Authors:** V. Pribula, L. P. Elek, T. Szekeres, T. A. Renko, P. Ruscsak, G. Vizin, X. Gonda

**Affiliations:** 1 Department of Psychiatry and Psychotherapy; 2 Department of Clinical Psychology; 3 Department of Internal Medicine and Oncology, Semmelweis University; 4 Institute of Psychology, Department of Clinical Psychology and Addictology, Budapest, Eotvos Lorand University, Budapest, Hungary

## Abstract

**Introduction:**

The concept of health is beyond the absence of disease; it encompasses the physical, mental, and social well-being of an individual, as well as their quality of life. Consequently, research into this multifaceted concept has become a crucial aspect of healthcare. With progress in medicine it has become increasingly evident that the primary objective of medical interventions is not merely to prolong life, but also to enhance its quality. A number of quality of life measures have been developed; however, the majority of these focus exclusively on physical health, and fail to fully encompass the dimensions that are crucial for the quality of life of individuals with mental health issues. The Mental Health Quality of Life (MHQoL) questionnaire, developed by Van Krugten et al., encompasses the seven most fundamental dimensions of mental health-related quality of life.

**Objectives:**

Our aim was to develop and test a Hungarian adaptation of the Mental Health Quality of Life (MHQoL) questionnaire and to compare the results in healthy subjects with those from individuals diagnosed with psychiatric disorders.

**Methods:**

Our ongoing research so far involved 189 participants, including 157 psychiatrically healthy subjects and 32 patients diagnosed with a mental disorder who completed the MHQoL as part of a larger test battery. Data were analysed using confirmatory factor analysis, reliability analysis and an independent samples t-test.

**Results:**

Confirmatory factor analysis showed that all items exhibited a satisfactory fit with the model. The factor weights for each item ranged from 0.45 to 0.79. MHQoL exhibited exemplary internal reliability, with Cronbach alpha index value of 0.81 and individual item-specific reliability coefficients ranging from 0.7 to 0.81. Independent samples t-tests showed significant differences between healthy and patient groups for items pertaining to future vision, mood, relationships and physical health, as well as when MHQoL mean total score and the mean scores of psychological well-being.

**Conclusions:**

Our preliminary results support that MHQoL is a suitable and reliable tool for assessing quality of life in relation to mental health, offering valuable insights into the domains in which psychiatric illnesses have the most significant impact on patients’ quality of life. The objective of our future research is to further validate the MHQoL questionnaire, including patients with different psychiatric diagnosis in order to contribute to the concept of healthcare that focuses not only on the elimination of symptoms but also on the improvement of quality of life, personalized to the needs of patients suffering from different psychiatric syndromes.

**Disclosure of Interest:**

None Declared

